# Supplementary material for: MetaRibo-Seq measures translation in microbiomes
Source: Nat Commun. 2020 Jun 29;11:3268. doi: 10.1038/s41467-020-17081-z (PMC7324362; doi:10.1038/s41467-020-17081-z)
Supplement: Supplementary file 10 — Supplementary Data 7 [file 41467_2020_17081_MOESM10_ESM.zip › File2/Confidence_High_Taxonomy/132456_out.krona.html]

Javascript must be enabled to view this page.

members
magnitude
magnitudeUnassigned
count
unassigned
taxon
rank

132456\_out

5

2
superkingdom
5

976
5
phylum

200643
class
5

171549
5
order

2005525
5
family

genus
5
375288


SRS011239\_contig\_number\_18901SRS020328\_contig\_number\_6332
2293113
species
2


SRS055966\_contig\_number\_962SRS063127\_contig\_number\_613
328812
species
2

species
1
387661

SRS056259\_contig\_number\_27990
